# Supplementary material for: Hippocampal ensemble dynamics and memory performance are modulated by respiration during encoding
Source: Nat Commun. 2023 Jul 27;14:4391. doi: 10.1038/s41467-023-40139-7 (PMC10374532; doi:10.1038/s41467-023-40139-7)
Supplement: Supplementary file 1 — Supplementary Information [file 41467_2023_40139_MOESM1_ESM.pdf]

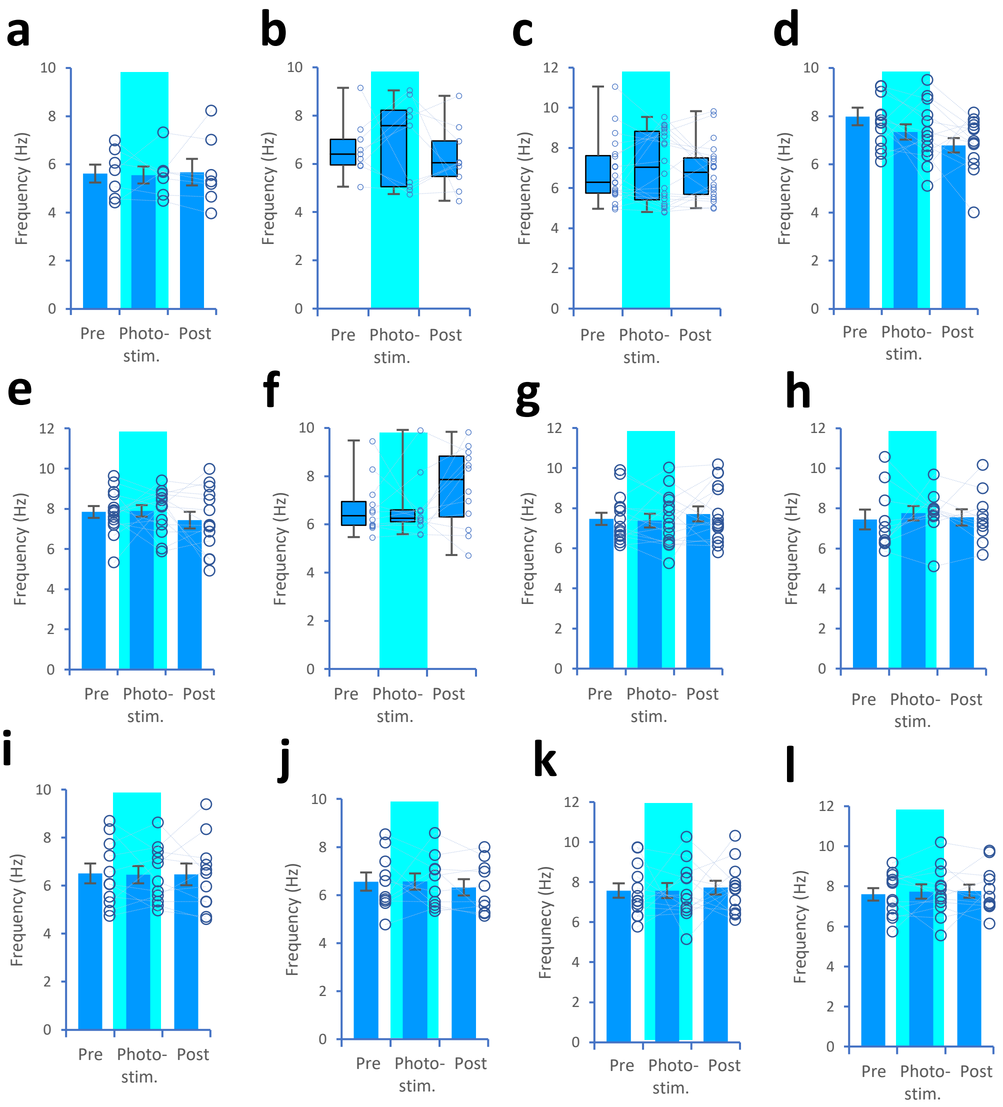

Supplementary Fig. 1 Regulation of breathing in individual *Vgat*-Cre$^-$ mice. Plots showing the frequency of whole-body plethysmographic signals among the preperiod (2 sec), photostimulation with blue light (465 nm, 2 sec), and postperiod (2 sec) in awake *Vgat*-Cre$^-$ mice at individual levels. **a-l.** There was no difference of the frequency in individual *Vgat*-Cre$^-$ mice (n = 7 trials, $F(2, 12) = 0.02$, $p = 0.98$, one-way repeated-measures ANOVA, **a**; n = 9 trials, $\chi^2(2) = 0$, $p = 1$, Friedman test, **b**; n = 20 trials, $\chi^2(2) = 2.24$, $p = 0.3$, **c**; n = 13 trials, $F(2, 24) = 2.64$, $p = 0.09$, **d**; n = 14 trials, $F(2, 26) = 0.61$ $p = 0.6$, **e**; n = 12 trials, $\chi^2(2) = 4.17$, $p = 0.12$, **f**; n = 15 trials, $F(2, 28) = 0.43$, $p = 0.7$. **g**; n = 10 trials, $F(2, 18) = 0.16$, $p = 0.9$, **h**; n = 11 trials, $F(2, 20) = 0.008$, $p = 0.99$, **i**; n = 10 trials, $F(2, 18) = 0.30$, $p = 0.7$, **j**; n = 13 trials, $F(2, 24) = 0.06$, $p = 0.9$, **k**; n = 12 trials, $F(2, 22) = 0.18$, $p = 0.8$, **l**). Three females (**a**, **b**, and **j**) and nine males (**c**, **d**, **e**, **f**, **g**, **h**, **I**, **k**, and **l**). Box plots indicate median, first, and third quantiles, and minimum and maximum values. Bar plots indicate means ± S.E.M. Circles in the graph represent individuals. Of note, the photostimulation with yellow light (595 nm) did not change respiration (data not shown).

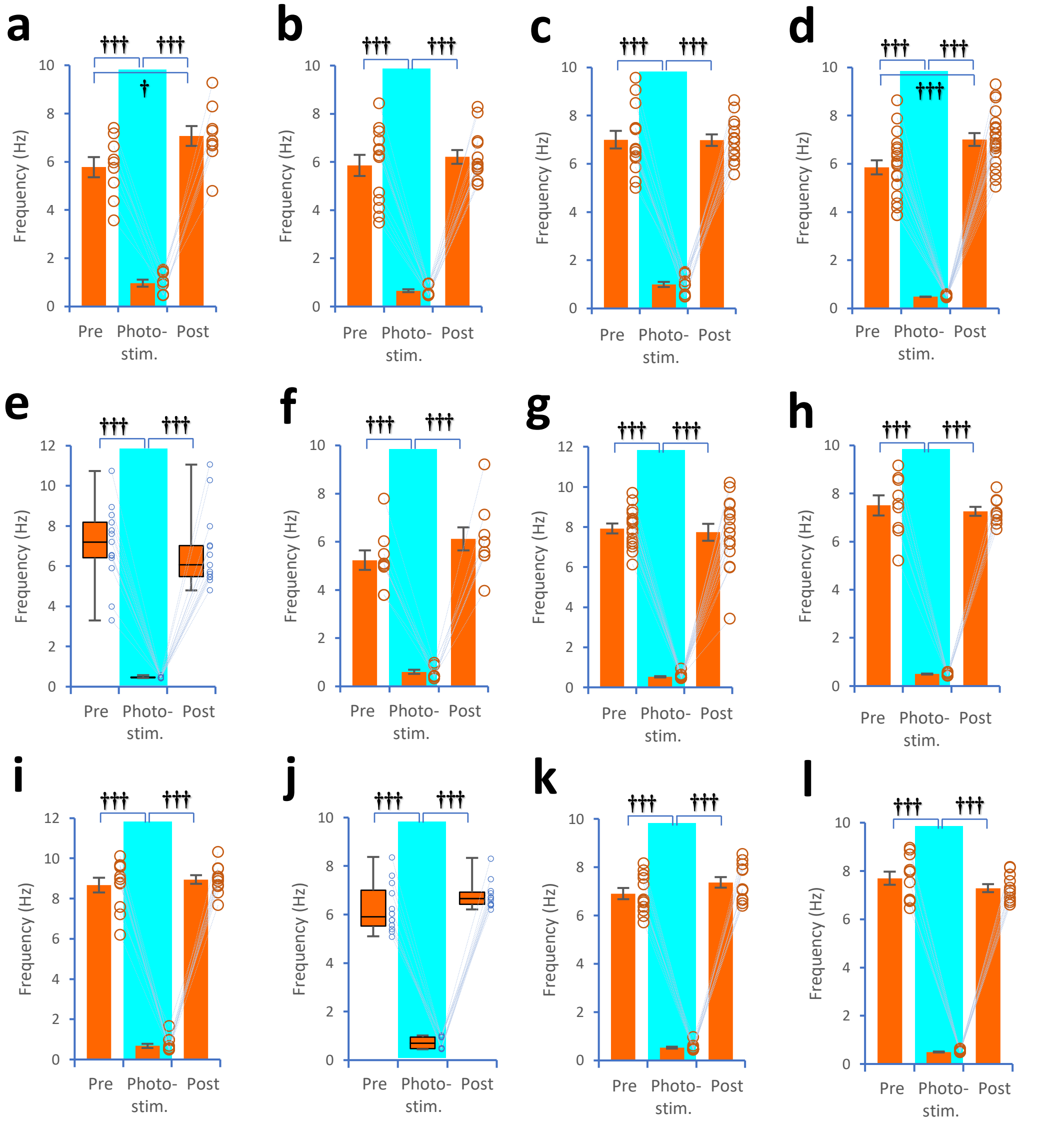

Supplementary Fig. 2 Regulation of breathing in individual *Vgat*-Cre$^+$ mice. Plots showing the frequency of whole-body plethysmographic signals among the preperiod (2 sec), photostimulation with blue light (465 nm, 2 sec), and postperiod (2 sec) in awake *Vgat*-Cre$^+$ mice at individual levels. **a-l.** In *Vgat*-Cre$^+$ mice, the frequency was remarkably decreased during the photostimulation compared to the pre- and postperiod (n = 9 trials, $F(2, 16) = 138$, $p = 7.8 \times 10^{-11}$, one-way repeated-measures ANOVA; pre vs. photo, $p = 7.4 \times 10^{-6}$, photo vs. post: $p = 7.3 \times 10^{-7}$, pre vs. post: $p = 0.02$, *post hoc* pairwise *t* test with two sided and Bonferroni correction, **a**; n = 13 trials, $F(2, 24) = 168$, $p = 7.8 \times 10^{-15}$, pre vs. photo, $p = 8.5 \times 10^{-8}$, photo vs. post: $p = 4.2 \times 10^{-10}$, pre vs. post: $p = 0.8$, **b**; n = 14 trials, $F(2, 26) = 218$, $p < 2.0 \times 10^{-16}$, pre vs. photo, $p = 3.5 \times 10^{-9}$, photo vs. post: $p = 2.6 \times 10^{-11}$, pre vs. post: $p = 1$, **c**; n = 20 trials, $F(2, 38) = 372$, $p < 2.0 \times 10^{-16}$, pre vs. photo, $p = 3.7 \times 10^{-13}$, photo vs. post: $p = 1.5 \times 10^{-15}$, pre vs. post: $p = 0.00008$, **d**; n = 13 trials, $\chi^2(2) = 19.8$, $p = 0.00005$, Friedman test, pre vs. photo, $p = 0.0007$, photo vs. post: $p = 0.0007$, pre vs. post: $p = 1$, *post hoc* pairwise Wilcoxon signed-rank test with two sided and Bonferroni correction, **e**; n = 9 trials, $F(2, 16) = 93.3$, $p = 1.5 \times 10^{-9}$, pre vs. photo, $p = 6.5 \times 10^{-6}$, photo vs. post: $p = 4.7 \times 10^{-6}$, pre vs. post: $p = 0.3$, **f**; n = 16 trials, $F(2, 30) = 234$, $p < 2.0 \times 10^{-16}$, pre vs. photo, $p = 2.6 \times 10^{-14}$, photo vs. post: $p = 1.2 \times 10^{-10}$, pre vs. post: $p = 1$, **g**; n = 9 trials, $F(2, 16) = 335$, $p = 8.8 \times 10^{-14}$, pre vs. photo, $p = 3.9 \times 10^{-7}$, photo vs. post: $p = 4.8 \times 10^{-10}$, pre vs. post: $p = 1$, **h**; n = 11 trials, $F(2, 20) = 332$, $p = 4.6 \times 10^{-16}$, pre vs. photo, $p = 5.5 \times 10^{-9}$, photo vs. post: $p = 1.8 \times 10^{-11}$, pre vs. post: $p = 1$, **i**; n = 12 trials, $\chi^2(2) = 19.5$, $p = 0.00006$, pre vs. photo, $p = 0.002$, photo vs. post: $p = 0.002$, pre vs. post: $p = 0.2$, **j**; n = 12 trials, $F(2, 22) = 545$, $p < 2.0 \times 10^{-16}$, pre vs. photo, $p = 8.2 \times 10^{-11}$, photo vs. post: $p = 2.6 \times 10^{-11}$, pre vs. post: $p = 0.2$, **k**; n = 11 trials, $F(2, 20) = 444$, $p < 2.0 \times 10^{-16}$, pre vs. photo, $p = 5.6 \times 10^{-10}$, photo vs. post: $p = 6.8 \times 10^{-12}$, pre vs. post: $p = 0.7$, **l**). Five females (**e**, **f**, **g**, **h**, and **l**) and seven males (**a**, **b**, **c**, **d**, **I**, **j**, and **k**). † $p < 0.05$ and ††† $p < 0.005$ (*post hoc* pairwise comparison). Box plots indicate median, first, and third quantiles, and minimum and maximum values. Bar plots indicate means ± S.E.M. Circles in the graph represent individuals. Of note, the photostimulation with yellow light (595 nm) did not change respiration (data not shown).

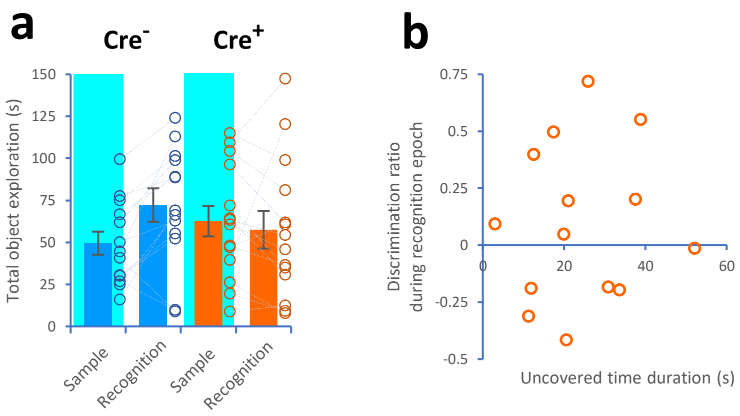

Supplementary Fig. 3 Object recognition memory using optogenetic manipulation. a. Bar plots showing the total object exploration time during the sample and recognition epochs for *Vgat*-Cre^{-} (Cre^{-}, n = 13, 7 females and 6 males, blue) and *Vgat*-Cre^{+} (Cre^{+}, n = 14, 10 females and 4 males, orange) mice (animal-type: $F(1, 25) = 0.005$, $p = 0.9$; epoch: $F(1, 25) = 2.14$, $p = 0.16$; interaction $F(1, 25) = 6.06$, $p = 0.02$, two-way mixed-design ANOVA). **b**. Plots showing correlations between the uncovered time duration of photostimulation during the sample epoch and the discrimination ratio during the recognition epoch in *Vgat*-Cre^{+} mice ($r = 0.10$, $t(12) = 0.34$, $p = 0.7$, Pearson's product-moment correlation). Cre^{-} mice (n = 13, 7 females and 6 males, blue) and Cre^{+} mice (n = 14, 10 females and 4 males, orange). Bar plots indicate means ± S.E.M. Circles in the graph represent individuals.

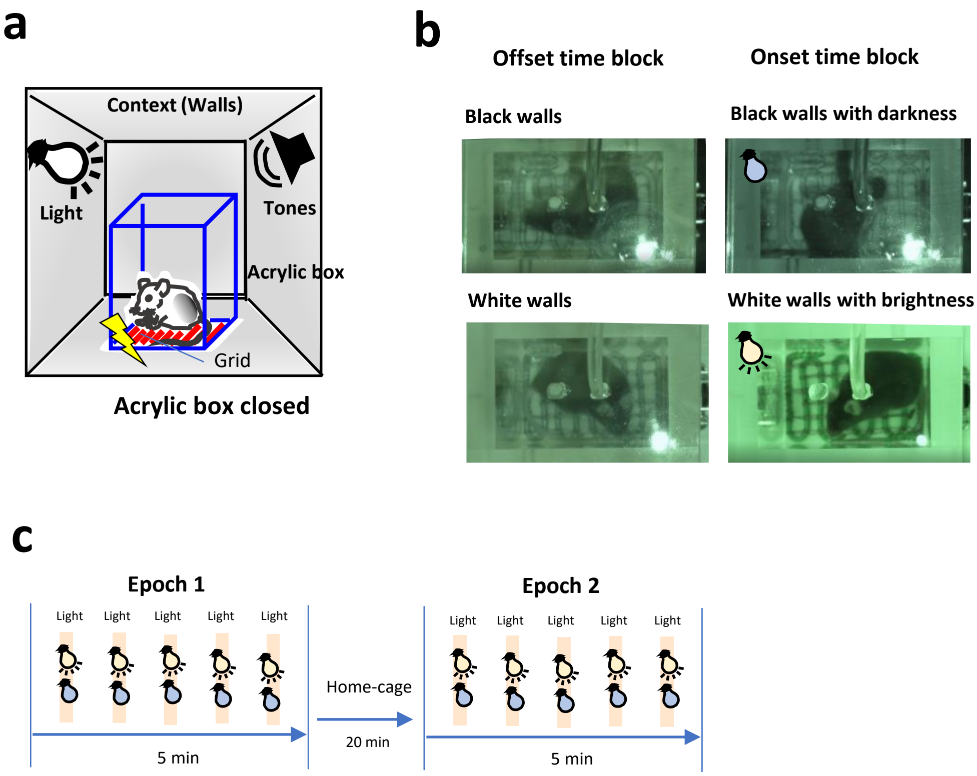

Supplementary Fig.4 Preliminary experiments of the fear conditioning paradigms (1). a, Drawings (Adapted from Pollak, D., Monje, F. & Lubec, G. The learned safety paradigm as a mouse model for neuropsychiatric research. Nat Protoc 5, 954–962, 2010)[33] showing a paradigm for the fear-conditioning task. Each animal was placed in a closed transparent acrylic box (see Methods). **b.** Images showing an animal in the acrylic box captured by an infrared video camera. The animals explored the inside of the box during the offset time block (natural light: 5 lx illuminance, left panels) and onset time block (bright light: 1,400 lx illuminance; or light off: 0 lx illuminance, right panels), and the box was surrounded by black walls (top panels) or white walls (bottom panels). The closed acrylic box was equipped with a whole-body plethysmography system to measure the cycle duration of respiration during motion (data not shown). **c.** The behavioral paradigm consisted of two 5-min epochs (Epochs 1 and 2) per day.

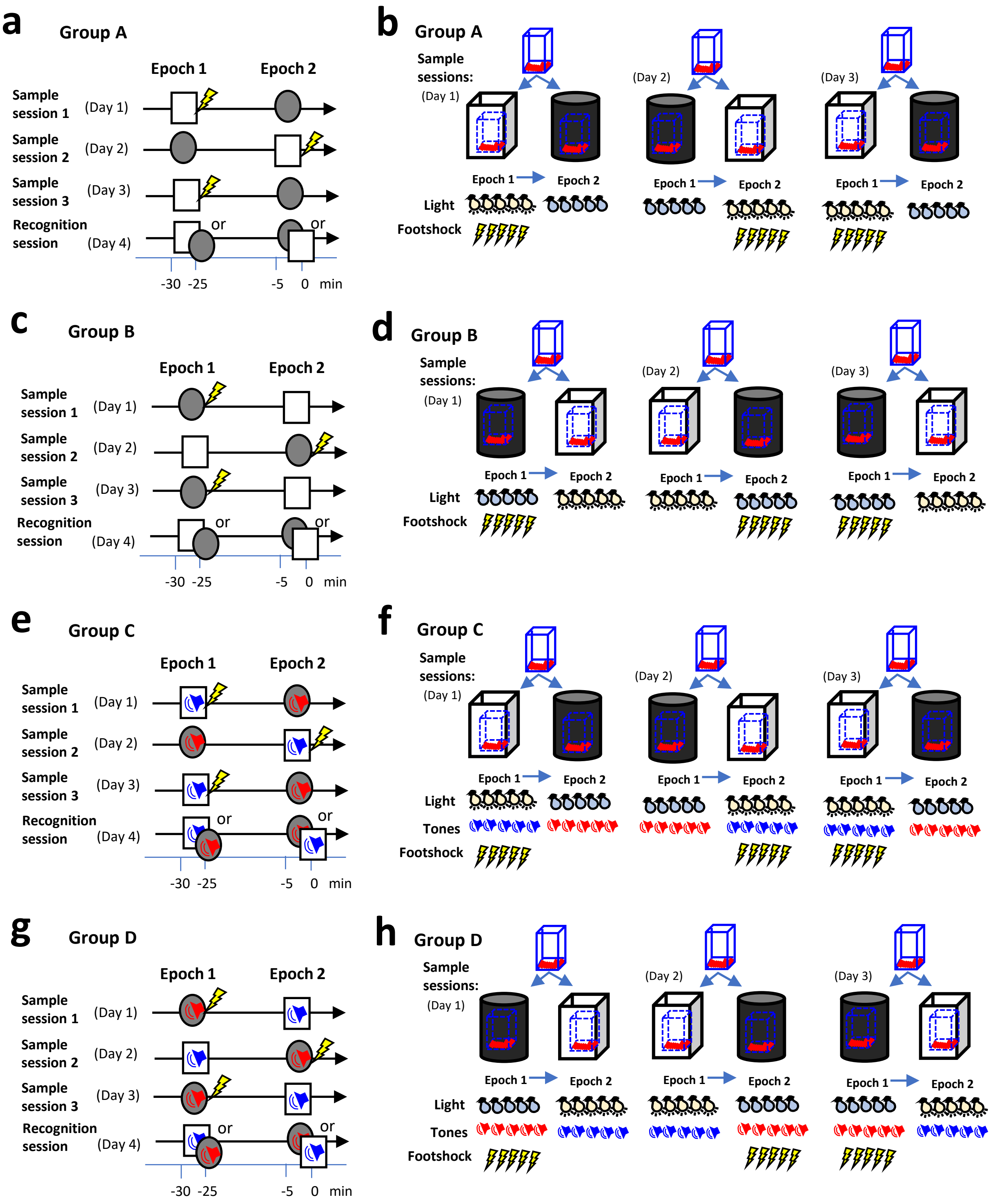

Supplementary Fig. 5 Preliminary experiments of the fear conditioning paradigms (2). On three consecutive days of the sample sessions, each animal was placed in the acrylic box, which was located away from either black or white walls. **a-d**, The animals in Group A (n = 3 males, **a,b**) learned black walls/darkness as the CS$^-$ and white walls/brightness as the CS$^+$, whereas the animals in Group B (n = 3 males, **c,d**) learned white walls/brightness as the CS$^-$ and black walls/darkness as the CS$^+$ (see Methods). **e-h**, We tested animals kept in the acrylic box with context/light-dependent CS$^+$/CS$^-$ in combination with auditory stimuli. The animals in Group C (n = 3 males, **e,f**) learned black walls/darkness/low tones as the CS$^-$ and white walls/brightness/high tones as the CS$^+$, whereas the animals in Group D (n = 3 males, **g,h**) learned white walls/brightness/high tones as the CS$^-$ and black walls/darkness/low tones as the CS$^+$ (see Methods).

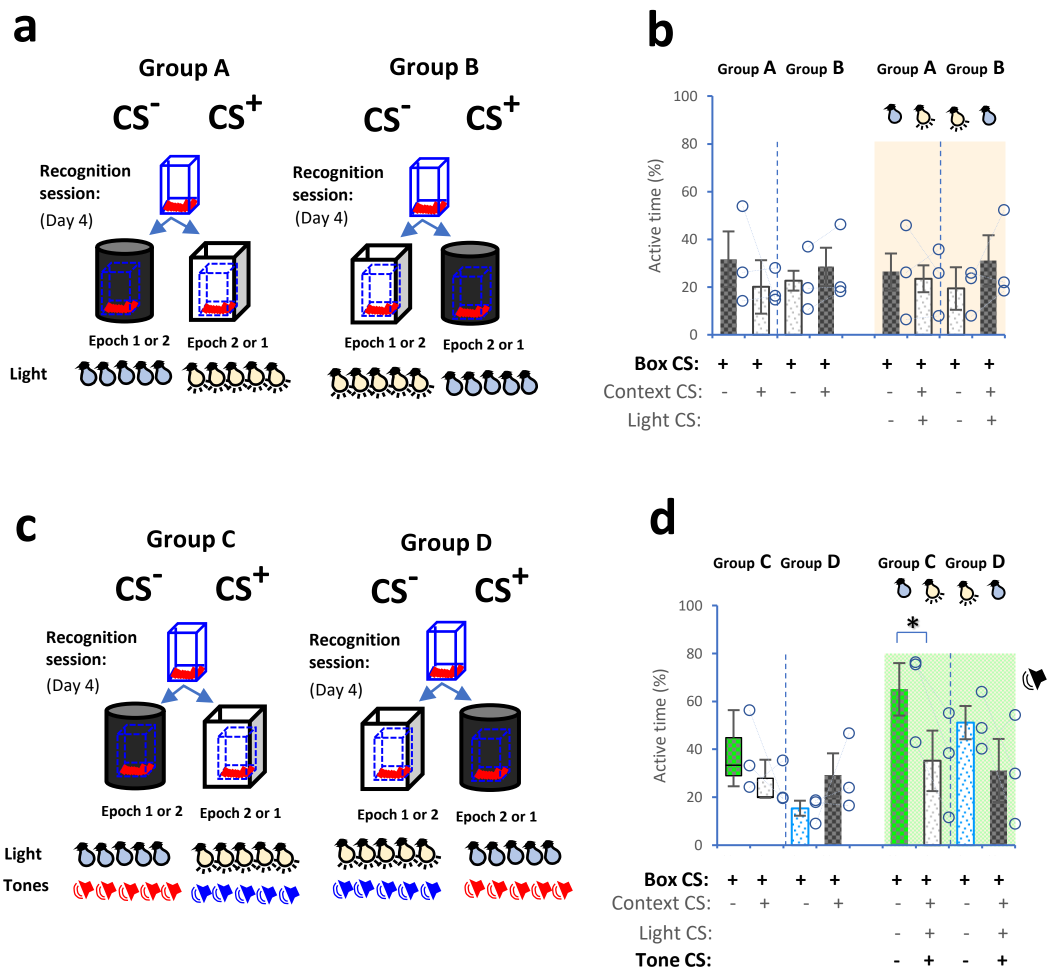

Supplementary Fig. 6 Preliminary experiments of the fear conditioning paradigms (3). a. Drawings showing a closed and transparent acrylic box surrounded by black and white walls in the contextual chamber for lights in Groups A (n = 3 males) and B (n = 3 males). Twenty-four hours after day 3 of the sample sessions, we tested the animals' ability to discriminate between the context/light-dependent CS$^-$ and context/light-dependent CS$^+$ during the recognition session. Each epoch included alternating offset and onset time blocks. **b.** Plots showing animals' temporal activity (active time) in the box during the offset and onset time blocks (light orange zone) between the context/light-dependent CS$^-$ and CS$^+$. No difference in active time was observed between the CS$^+$ and CS$^-$, or between color walls (black vs. white) during the offset time blocks (Group A: $t(2) = 0.85$, $p = 0.5$; Group B: $t(2) = 2.15$, $p = 0.16$, two-tailed paired *t* test) and onset time blocks (Group A: $t(2) = 0.34$, $p = 0.8$; Group B: $t(2) = 1.26$, $p = 0.3$). These results indicated that the animals in Groups A and B maintained freezing behavior during the presentation of the context/light-dependent CS$^+$ and CS$^-$ during the offset and onset time blocks. **c.** We tested the animals' ability to discriminate between context/light/tone-dependent CS$^-$ and context/light/tone-dependent CS$^+$ in Groups C (n = 3 males) and D (n = 3 males). **d.** Plots showing active time in the box between the context/light/tone-dependent CS$^-$ and CS$^+$ during the offset (Group C: $p = 0.5$, two-sided Wilcoxon signed rank test; Group D: $t(2) = 1.15$, $p = 0.4$, two-tailed paired *t* test) and onset (Group C: $t(2) = 5.80$, $p = 0.03$; Group D: $t(2) = 1.17$, $p = 0.4$) time blocks (light green zone). The active time was higher for the combined CS$^-$ than for the combined CS$^+$ in Group C. These results revealed that the context, light, and tones were all associated with footshocks as the CS$^+$/CS$^-$, resulting in overriding the box-dependent CS$^+$ (i.e., acrylic box). * $p < 0.05$ (two-tailed paired *t* test). Box plots indicate median, first, and third quantiles, and minimum and maximum values. Bar plots indicate means ± S.E.M. Circles in the graph represent individuals.

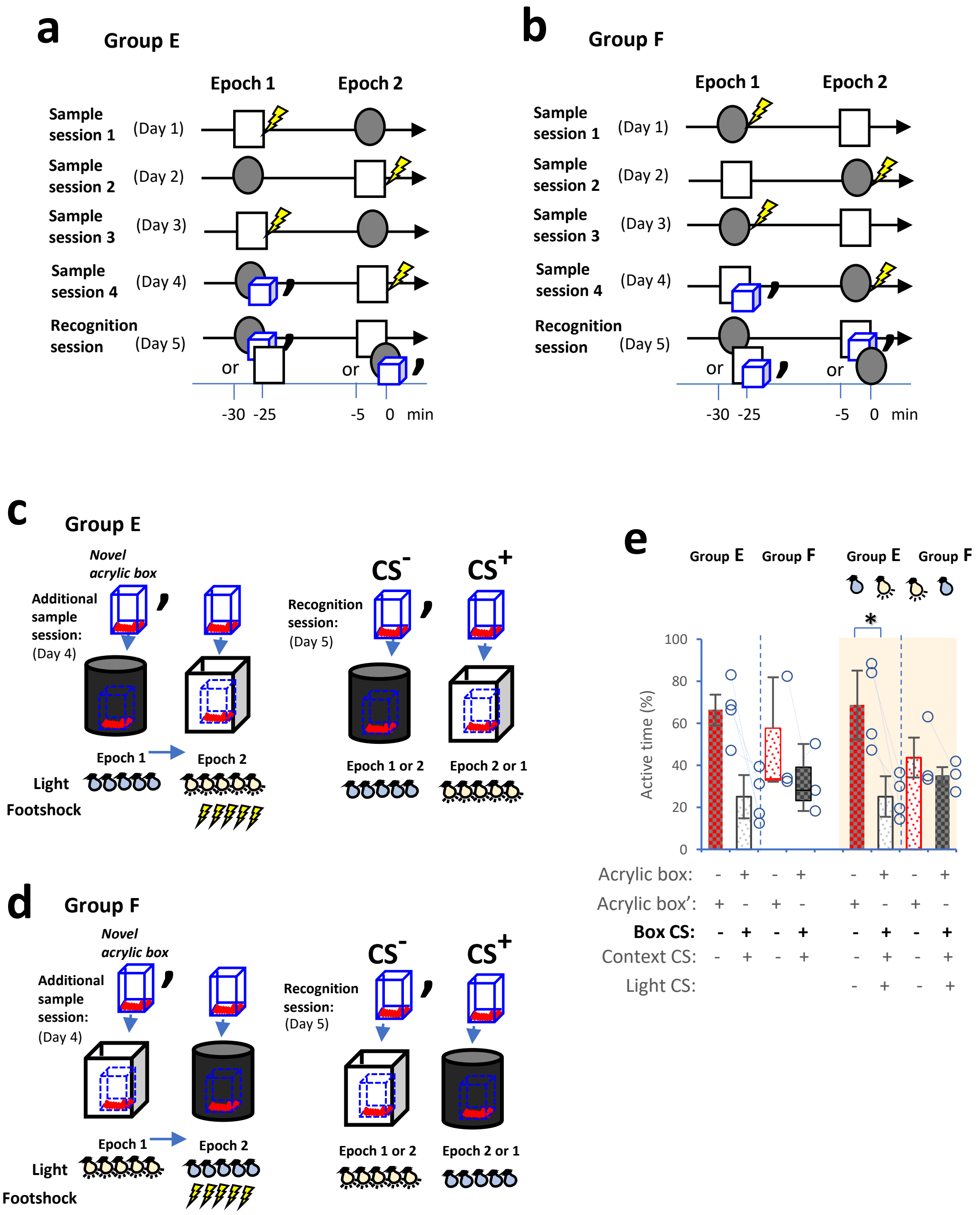

Supplementary Fig. 7 Preliminary experiments of the fear conditioning paradigms (4). a-d. During an additional sample session (day 4), the animals in Group E (n = 4 males, **a,c**) and Group F (n = 3 males, **b,d**) were placed in a novel acrylic box surrounded by context/light-dependent CS$^-$, and their ability to distinguish between context/light-dependent CS$^+$ and CS$^-$ during the recognition session was tested (day 5, see Methods). **e.** Plots showing active time in the box between the context/light-dependent CS$^-$ and CS$^+$ during the offset (Group E: $t(3) = 3.10$, $p = 0.053$, two-tailed paired *t* test; Group F: $p = 0.75$, two-sided Wilcoxon signed rank test) and onset (Group E: $t(3) = 4.09$, $p = 0.03$; Group F: $t(2) = 0.87$, $p = 0.5$, two-tailed paired *t* test) time blocks (light orange zone). The active time was higher for the combined CS$^-$ than for the combined CS$^+$ in Group E. Thus, the acrylic box was strongly associated with footshocks as a box-dependent CS$^+$ rather than a context/light-dependent CS$^+$/CS$^-$ when the animals were placed in the same acrylic box. * $p < 0.05$ (two-tailed paired *t* test). Box plots indicate median, first, and third quantiles, and minimum and maximum values. Bar plots indicate means ± S.E.M. Circles in the graph represent individuals.

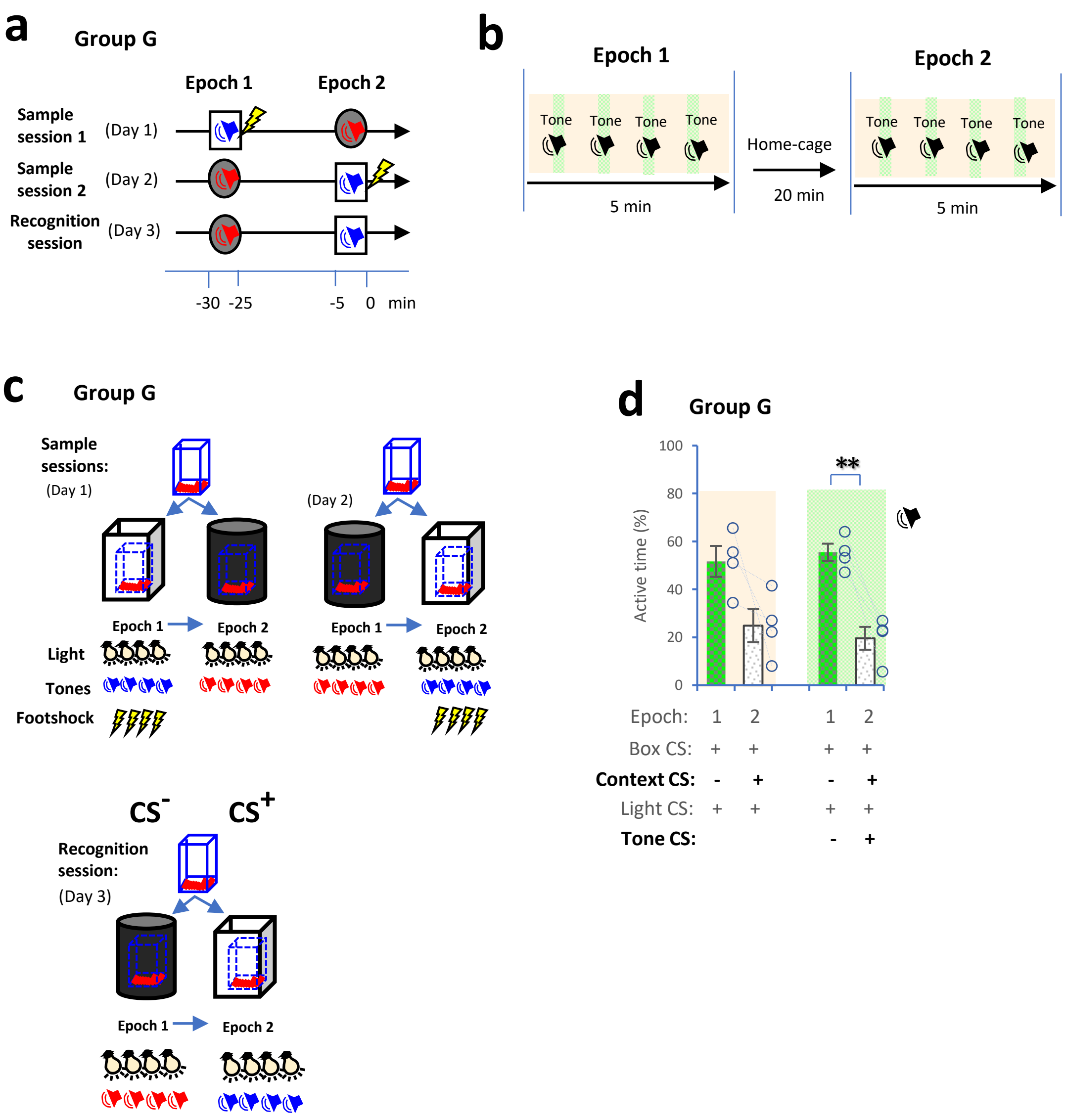

Supplementary Fig.8 Preliminary experiments of the fear conditioning paradigms (5). a,b. The paradigm of Group G consisted of two-day sample sessions and a one-day recognition session (modified by the Group C paradigm). Each epoch alternated between offset (light orange zone) and onset (tones, light green zone) time blocks under bright light. **c**, The animals in Group G (n = 4 males) learned white walls/high tones as the CS^{+} and black walls/low tones as the CS^{-} (see Methods). **d.** Bar plots showing active time in the box during the offset (light orange zone) and onset (light green zone) time blocks between the context/tone-dependent CS^{-} and CS^{+}. No difference was found in the active time between the CS^{-} and CS^{+} during the offset blocks ($t(3) = 2.30$, $p = 0.11$, two-tailed paired t test), whereas the active time was lower for the CS^{+} than for the CS^{-} during the onset blocks ($t(3) = 6.60$, $p = 0.007$). ** $p < 0.01$ (two-tailed paired t test). Bar plots indicate means ± S.E.M. Circles in the graph represent individuals.

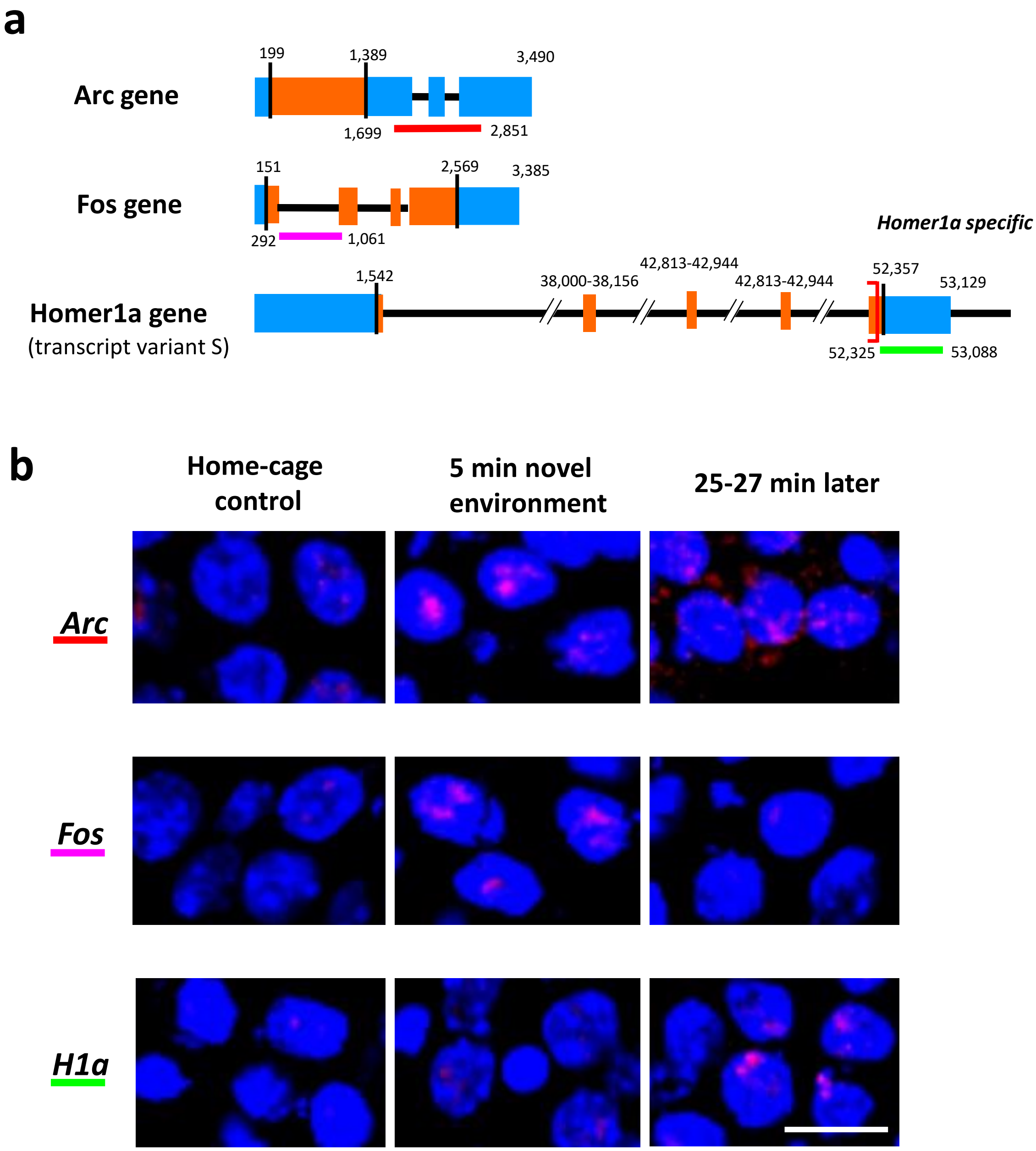

Supplementary Fig. 9 Designs of RNA probes and distribution of expression patterns for *Arc*, *Fos*, and *Homer1a* (*H1a*). a. Drawings showing coding regions (orange box) and untranslated regions (blue box) with introns (black line) of mouse genes for *Arc* (NCBI RefSeq: NC_000081.7), *Fos* (NC_000078.7), and *Homer1* transcript variant S (Homer1a or H1a, NC_000079.7). Selected sequences for each RNA probe are underlined (*Arc*: red line; *Fos*: pink line; *H1a*: green line). **b.** Images showing spatiotemporal expression patterns of cells (DAPI stained, blue) for *Arc* (upper panels), *Fos* (middle horizontal panels),and *H1a* (bottom panels) in the distal part of CA3 in the dorsal hippocampus of wild-type mice. Three animal conditions, home-cage control (left panels), novel environment exposure for 5 min (middle vertical panels), and 25-27 min following the exposure (right panels). The scale bar is 20 μm in **b**.

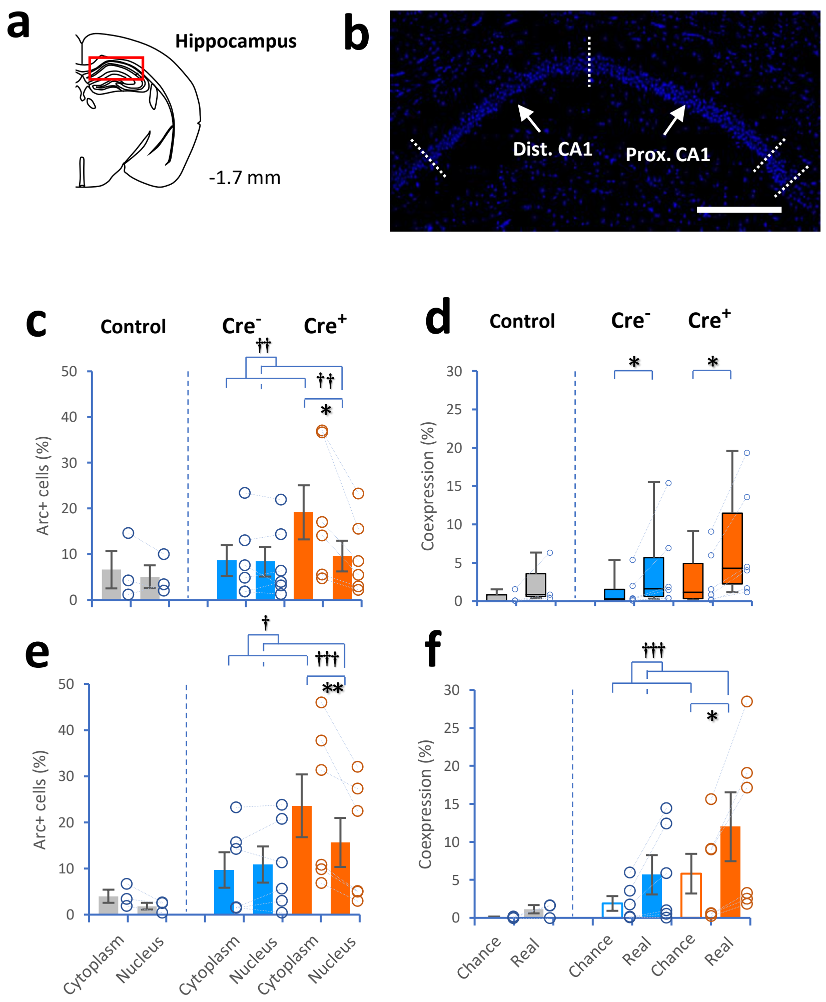

Supplementary Fig. 10 *Arc* catFISH methods for assessing CA1 cell ensembles during the conditioning task. a,b. Drawings (Adapted from Paxinos, G., & Franklin., K. B. J: The mouse brain in stereotaxic coordinates, 2nd ed. Academic Press, San Diego, CA, 2001)[60] and images showing the hippocampus (**a**) and CA1 with DAPI-stained nuclei (blue, **b**). **c,d.** Plots showing the proportion of *Arc*-positive cells in distal CA1 in home-cage control (n = 3, 1 female and 2 males, gray), *Vgat*-Cre$^-$ (Cre$^-$, n = 6, 2 females and 4 males, blue), and *Vgat*-Cre$^+$ (Cre$^+$, n = 6, 2 females and 4 males, orange) mice in the conditioning task (**c**, cytoplasm vs. nucleus in animal-type: $F(1, 10) = 1.09$, $p = 0.3$; distribution: $F(1, 10) = 10.14$, $p = 0.0098$; interaction: $F(1, 10) = 9.25$, $p = 0.012$, two-way mixed-design ANOVA; Cre$^-$ vs. Cre$^+$ in cytoplasm: $t(7.95) = 1.55$, $p = 0.2$, two-tailed Welch t test; nucleus: $t(9.99) = 0.26$, $p = 0.8$; cytoplasm vs. nucleus in Cre$^-$: $t(5) = 0.22$, $p = 0.8$, two-tailed paired t test; Cre$^+$: $t(5) = 3.29$, $p = 0.02$; **d**, Cre$^-$ vs. Cre$^+$ in coexpression: $p = 0.4$, two-sided Mann-Whitney U test; chance vs. real in Cre$^-$: $p = 0.03$, two-sided Wilcoxon signed-rank test; Cre$^+$: $p = 0.03$). **e,f.** Bar plots showing the proportion of *Arc*-positive cells in proximal CA1 (**e**, cytoplasm vs. nucleus in animal-type: $F(1, 10) = 1.72$, $p = 0.2$; distribution: $F(1, 10) = 7.53$, $p = 0.02$; interaction: $F(1, 10) = 13.76$, $p = 0.004$; Cre$^-$ vs. Cre$^+$ in cytoplasm: $t(7.87) = 1.78$, $p = 0.11$; nucleus: $t(9.24) = 0.72$, $p = 0.5$; cytoplasm vs. nucleus in Cre$^-$: $t(5) = 0.97$, $p = 0.4$; Cre$^+$: $t(5) = 3.72$, $p = 0.014$; **f**, chance vs. real coexpression in animal-type: $F(1, 10) = 1.65$, $p = 0.2$; coexpression: $F(1, 10) = 15.30$, $p = 0.003$; interaction: $F(1, 10) = 0.90$, $p = 0.4$; Cre$^-$ vs. Cre$^+$ in coexpression: $t(7.96) = 1.21$, $p = 0.3$; chance vs. real in Cre$^-$: $t(5) = 2.29$, $p = 0.07$; Cre$^+$: $t(5) = 3.19$, $p = 0.02$). A scale bar in **b** is 300 µm. Home-cage control mice (n = 3, 1 female and 2 males, gray), Cre$^-$ mice (n = 6, 2 females and 4 males, blue) and Cre$^+$ mice (n = 6, 2 females and 4 males, orange) in **c-f**. † $p < 0.05$, †† $p \le 0.01$, and ††† $p < 0.005$ (main effects and interactions using ANOVA). * $p < 0.05$ and ** $p \le 0.01$ (two-tailed Welch t test, two-tailed paired t test, two-sided Mann-Whitney U test, and two-sided Wilcoxon signed-rank test). Box plots indicate median, first, and third quantiles, and minimum and maximum values. Bar plots indicate means ± S.E.M. Circles in the graph represent individuals.

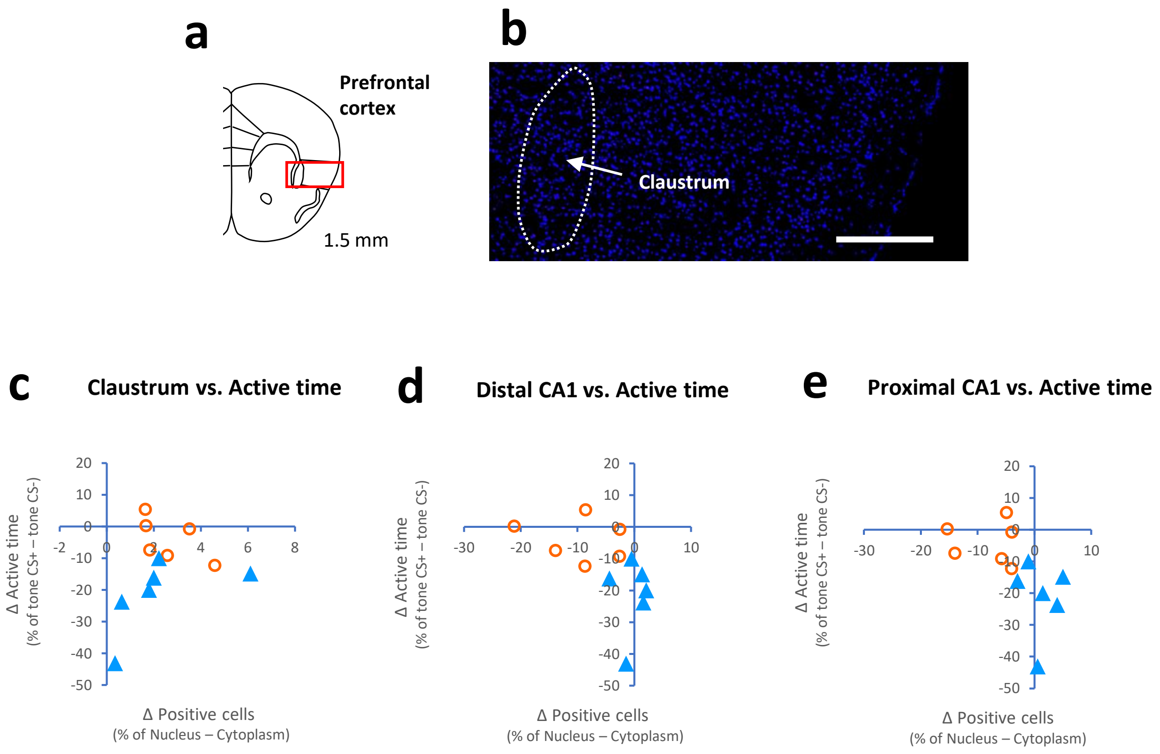

Supplementary Fig. 11 Correlation between neuronal activation and behavioral factors. a,b. Drawings (Adapted from Paxinos, G., & Franklin., K. B. J: The mouse brain in stereotaxic coordinates, 2nd ed. Academic Press, San Diego, CA, 2001)[60] and images showing the claustrum (**a**) with DAPI-stained nuclei (**b**). The claustrum has strong afferent and effect projections to the ACC (see Methods). **c-e.** There was no correlation between changes in active time (tone CS$^+$ – tone CS$^-$) and changes in the proportion of *Arc*-positive cells (nucleus – cytoplasm) in the claustrum (r = 0.30, t(10) = 0.98, p = 0.4, Pearson's product-moment correlation), distal CA1 (r = -0.52, t(10) = 1.95, p = 0.08, Pearson's product-moment correlation), or proximal CA1 (r = -0.57, t(10) = 2.19, p = 0.053) in *Vgat*-Cre$^-$ (Cre$^-$, blue), and *Vgat*-Cre$^+$ (Cre$^+$, orange) mice. The scale bar is 400 μm in **b**. Circles and triangles in the graph represent individuals.

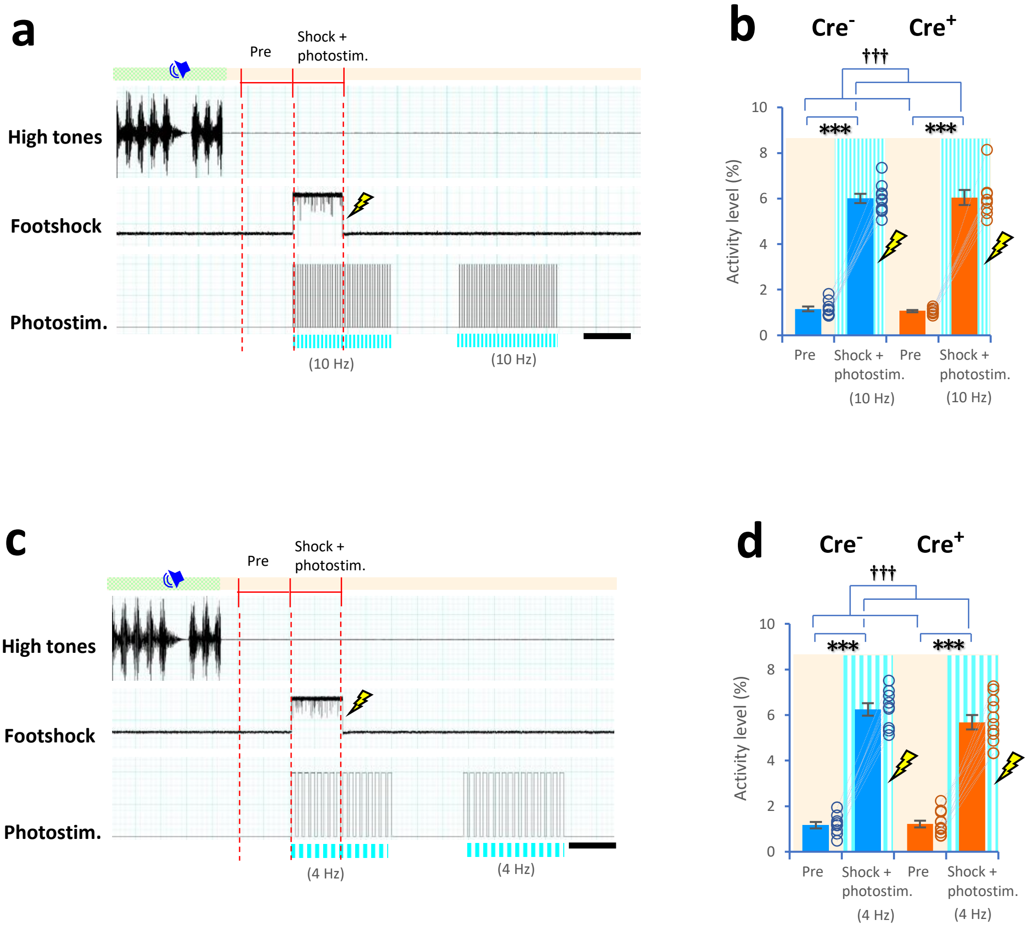

Supplementary Fig. 12 Activity levels induced by footshock with optogenetic manipulation. a,b. Plots showing time courses and activity levels in the acrylic box during the periods of the prestimulation and footshock with 10-Hz photostimulation in Cre$^-$ (n = 10, 8 females and 2 males) and Cre$^+$ (n = 8, 5 females and 3 males) mice (animal type: $F(1, 16) = 0.02$, $p = 0.9$; period: $F(1, 16) = 642.7$, $p = 2.4 \times 10^{-14}$; interaction: $F(1, 16) = 0.11$, $p = 0.7$, two-way mixed-design ANOVA; prestimulation vs. footshock: Cre$^-$: $t(9) = 21.33$, $p = 5.2 \times 10^{-9}$; Cre$^+$: $t(7) = 15.04$, $p = 1.4 \times 10^{-6}$, two-tailed paired *t* test; Cre$^-$ vs. Cre$^+$ in footshock: $t(12.00) = 0.09$, $p = 0.9$, two-tailed Welch's *t* test). **c,d.** Plots showing time courses and activity levels during the periods of the prestimulation and footshock with 4-Hz photostimulation in Cre$^-$ (n = 9, 4 females and 5 males) and Cre$^+$ (n = 11, 7 females and 4 males) mice (animal type: $F(1, 18) = 0.89$, $p = 0.4$; period: $F(1, 18) = 591.4$, $p = 3.2 \times 10^{-15}$; interaction: $F(1, 18) = 2.40$, $p = 0.14$; prestimulation vs. footshock: Cre$^-$: $t(8) = 20.94$, $p = 2.8 \times 10^{-8}$; Cre$^+$: $t(10) = 15.24$, $p = 3.0 \times 10^{-8}$; Cre$^-$ vs. Cre$^+$ in footshock: $t(18.00) = 1.35$, $p = 0.2$). The scale bars are 2 s in **a,c**. ††† $p < 0.005$ (main effects and interactions using ANOVA). *** $p < 0.005$ (two-tailed paired *t* test). Bar plots indicate means ± S.E.M. Circles in the graph represent individuals.
