# Supplementary material for: Hippocampal ensemble dynamics and memory performance are modulated by respiration during encoding
Source: Nat Commun. 2023 Jul 27;14:4391. doi: 10.1038/s41467-023-40139-7 (PMC10374532; doi:10.1038/s41467-023-40139-7)
Supplement: Supplementary file 3 — Reporting Summary [file 41467_2023_40139_MOESM3_ESM.pdf]

Corresponding author(s): Nozomu Nakamura

Last updated by author(s): Jun 15, 2023

Reporting Summary

Nature Portfolio wishes to improve the reproducibility of the work that we publish. This form provides structure for consistency and transparency in reporting. For further information on Nature Portfolio policies, see our Editorial Policies and the Editorial Policy Checklist.

Statistics

For all statistical analyses, confirm that the following items are present in the figure legend, table legend, main text, or Methods section.

n/a	Confirmed	
☐	☒	The exact sample size (*n*) for each experimental group/condition, given as a discrete number and unit of measurement
☐	☒	A statement on whether measurements were taken from distinct samples or whether the same sample was measured repeatedly
☐	☒	The statistical test(s) used AND whether they are one- or two-sided<br>*Only common tests should be described solely by name; describe more complex techniques in the Methods section.*
☒	☐	A description of all covariates tested
☐	☒	A description of any assumptions or corrections, such as tests of normality and adjustment for multiple comparisons
☐	☒	A full description of the statistical parameters including central tendency (e.g. means) or other basic estimates (e.g. regression coefficient) AND variation (e.g. standard deviation) or associated estimates of uncertainty (e.g. confidence intervals)
☐	☒	For null hypothesis testing, the test statistic (e.g. *F*, *t*, *r*) with confidence intervals, effect sizes, degrees of freedom and *P* value noted<br>*Give P values as exact values whenever suitable.*
☒	☐	For Bayesian analysis, information on the choice of priors and Markov chain Monte Carlo settings
☒	☐	For hierarchical and complex designs, identification of the appropriate level for tests and full reporting of outcomes
☐	☒	Estimates of effect sizes (e.g. Cohen's *d*, Pearson's *r*), indicating how they were calculated

Our web collection on statistics for biologists contains articles on many of the points above.

Software and code

Policy information about availability of computer code

Data collection	Data of respiration, photostimulaton pulses , tones, footshocks, and electrocardiogram: Respiratory flow heads, Spirometer, PowerLab, and LabChart 8.1 (AD Instruments); Data of exploratory and freezing behavior: 60 Hz frame infrared video camera (HC-W870M, Panasonic, Japan); Data of fluorescent images: Laser scanning confocal microscopy (LSM 780, Carl Zeiss).
Data analysis	Data of respiration and electrocardiogram: R version 3.6.1 software (https://www.R-project.org/); Data of exploratory and freezing behavior: Image J 1.51k (NIH, http//:imagej.nih.gov/ij), MATLAB R2018b with the computer vision toolbox and image processing toolbox (MathWorks), and R version 3.6.1 software (https://www.R-project.org/); Data of fluorescent images: MATLAB R2021b with the image processing toolbox (MathWorks) and R version 3.6.1 software (https://www.R-project.org/).

For manuscripts utilizing custom algorithms or software that are central to the research but not yet described in published literature, software must be made available to editors and reviewers. We strongly encourage code deposition in a community repository (e.g. GitHub). See the Nature Portfolio guidelines for submitting code & software for further information.

Data

Policy information about availability of data

All manuscripts must include a data availability statement. This statement should provide the following information, where applicable:
- Accession codes, unique identifiers, or web links for publicly available datasets
- A description of any restrictions on data availability
- For clinical datasets or third party data, please ensure that the statement adheres to our policy

The data that support the main findings of this study are available from the corresponding author upon request. The datasets used in this study are available at: https://github.com/nakamunh/Nakamura_2023_Nat_Commun Source data are provided with this paper.

Human research participants

Policy information about studies involving human research participants and Sex and Gender in Research.

Reporting on sex and gender	N/A
Population characteristics	N/A
Recruitment	N/A
Ethics oversight	N/A

Note that full information on the approval of the study protocol must also be provided in the manuscript.

Field-specific reporting

Please select the one below that is the best fit for your research. If you are not sure, read the appropriate sections before making your selection.

☒ Life sciences ☐ Behavioural & social sciences ☐ Ecological, evolutionary & environmental sciences

For a reference copy of the document with all sections, see nature.com/documents/nr-reporting-summary-flat.pdf

Life sciences study design

All studies must disclose on these points even when the disclosure is negative.

Sample size	Sample sizes for animals were based on comparable n-values from the literature published previously (see ref. 25, 26, 27).
Data exclusions	All animals got the virus injection and implantation into the PreBötzinger complex in the ventrolateral medulla. The animals with insufficient quality of breathing rhythm in the awake state were excluded in this study. No data that were applicable for each analysis were excluded.
Replication	Behavioral experimental settings of fear conditioning, i.e., footshocks, tones, and photostimulation, were used as replicates independently in all animals. Experiments of in situ hybridization were repeated at least twice independently and the results are reproducible. Statistically significant results were replicated at least three times in all experiments.
Randomization	All animals were randomly assigned to the experimental groups in this study. Animals in the experimental groups (Cre- vs. Cre+ mice) tested their behavior in a randomized order on the same day.
Blinding	The virus injections were performed with the investigator blind to animals. For cell counting, allocation during data collection was performed blind to the experimental groups. Blinding is not relevant to the groups of animals in behavioral experiments because physiological differences (e.g., apnea) would be present during the experiment. To avoid biased criteria of judgment, all data collection and all data analysis was automatically collected and performed with constant settings and thresholds.

Reporting for specific materials, systems and methods

We require information from authors about some types of materials, experimental systems and methods used in many studies. Here, indicate whether each material, system or method listed is relevant to your study. If you are not sure if a list item applies to your research, read the appropriate section before selecting a response.

Materials & experimental systems

n/a	Involved in the study
☐	☒ Antibodies
☒	☐ Eukaryotic cell lines
☒	☐ Palaeontology and archaeology
☐	☒ Animals and other organisms
☒	☐ Clinical data
☒	☐ Dual use research of concern

Methods

n/a	Involved in the study
☒	☐ ChIP-seq
☒	☐ Flow cytometry
☒	☐ MRI-based neuroimaging

Antibodies

Antibodies used	Anti-Digoxigenin-POD antibody (Fab fragments from sheep, 11207733910, Roche Diagnostics), anti-fluorescein-POD antibody (Fab fragments from sheep, 11426346910, Roche Diagnostics)
Validation	These antibodies have been validated by the suppliers and have been cited many times as listed on the manufacture's website as well as our papers with relevant images (Nakamura et al., Neuroendocrinology 80: 308, 2004; Nakamura et al., Neuroscience 166: 994, 2010; Nakamura et al., J Neurosci 33: 115, 2013; Nakamura et al., Hippocampus 26: 67, 2016).

Animals and other research organisms

Policy information about studies involving animals; ARRIVE guidelines recommended for reporting animal research, and Sex and Gender in Research

Laboratory animals	Adult Vgat-Cre mice (males and females, B6J.129S6(FVB)-Slc32a1<tm2(cre)Low1>MwarJ, JAX Mice 016962 B6J background, 028862, Jackson Laboratory) and adult wild-type C57BL/6J mice (males and females, Japan Charles River) were housed with food and water available ad libitum under a 12-h light cycle in a temperature-controlled room (23 ± 1 °C and 50 ± 1% humidity) at a minimum of 90% of normal body weight. The virus injections were carried on 12-14 weeks of age for mice.
Wild animals	No wild animals were used.
Reporting on sex	Experiments were performed in both female and male mice: Object recognition memory task (Cre- mice n = 13, 6 females and 7 males; Cre+ mice n = 14, 10 females and 4 males), fear conditioning task using flat photostimulation (Cre- mice n = 11, 3 females and 8 males; Cre+ mice n = 11, 4 females and 7 males), fear conditioning task using 10 Hz photostimulation (Cre- mice n = 10, 8 females and 2 males; Cre+ mice n = 8, 5 females and 3 males), and fear conditioning task using 4 Hz photostimulation (cre- mice n = 9, 4 females and 5 males; Cre+ mice n = 11, 7 females and 4 males).
Field-collected samples	This study did not require samples collected from the field.
Ethics oversight	All animal procedures were performed in accordance with the Guidelines for Proper Conduct of Animal Experiments, Science Council of Japan, and the regulations for animal experimentation of the Hyogo Medical University and were approved by the Animal Experiment Committee and the Ethical Committee at the Hyogo Medical Univerisity (18-039, 19-005, 20-049, 218001, HCM-0921).

Note that full information on the approval of the study protocol must also be provided in the manuscript.
